# Design, Synthesis and Biological Evaluation of Novel Inhibitors of *Trypanosoma brucei* Pteridine Reductase 1

**DOI:** 10.1002/cmdc.201000450

**Published:** 2010-11-25

**Authors:** Daniel Spinks, Han B Ong, Chidochangu P Mpamhanga, Emma J Shanks, David A Robinson, Iain T Collie, Kevin D Read, Julie A Frearson, Paul G Wyatt, Ruth Brenk, Alan H Fairlamb, Ian H Gilbert

**Affiliations:** Drug Discovery Unit, Division of Biological Chemistry & Drug Discovery, College of Life Sciences, University of Dundee, Sir James Black CentreDundee, Scotland, DD1 5EH (UK)

**Keywords:** antiprotozoal agents, drug discovery, pteridine reductase, structure-based drug design, *Trypanosoma brucei*

## Abstract

Genetic studies indicate that the enzyme pteridine reductase 1 (PTR1) is essential for the survival of the protozoan parasite *Trypanosoma brucei*. Herein, we describe the development and optimisation of a novel series of PTR1 inhibitors, based on benzo[*d*]imidazol-2-amine derivatives. Data are reported on 33 compounds. This series was initially discovered by a virtual screening campaign (*J. Med. Chem*., **2009**, *52*, 4454). The inhibitors adopted an alternative binding mode to those of the natural ligands, biopterin and dihydrobiopterin, and classical inhibitors, such as methotrexate. Using both rational medicinal chemistry and structure-based approaches, we were able to derive compounds with potent activity against *T. brucei* PTR1 (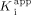
=7 nm), which had high selectivity over both human and *T. brucei* dihydrofolate reductase. Unfortunately, these compounds displayed weak activity against the parasites. Kinetic studies and analysis indicate that the main reason for the lack of cell potency is due to the compounds having insufficient potency against the enzyme, which can be seen from the low *K*_m_ to *K*_i_ ratio (*K*_m_=25 nm and *K*_i_=2.3 nm, respectively).

## Introduction

Human African trypanosomiasis (HAT) is a serious health problem in sub-Saharan Africa, with an estimated 50 000 new infections each year, and over 60 million people in 36 countries are at risk of infection.^1^ HAT is a progressive and ultimately fatal disease. The causative agents of HAT are the protozoan parasites *Trypanosoma brucei gambiense* and *T. b. rhodesiense*, which are transmitted by the bite of a tsetse fly. In the initial stage of the infection, parasites multiply in the blood and lymphatic systems of the host, causing fever, headaches and joint pains. Eventually they cross the blood–brain barrier (BBB) to invade the central nervous system (CNS). Once the infection penetrates the CNS, it is very difficult to treat, causing the classical symptoms of mental deterioration leading to coma and death, which give the disease its more commonly recognisable name of ‘sleeping sickness’. The current drugs to treat HAT are inadequate, due to poor efficacy, side effects and the requirement for parenteral administration, which is not appropriate for a rural African setting.^2^

**Figure 1:**
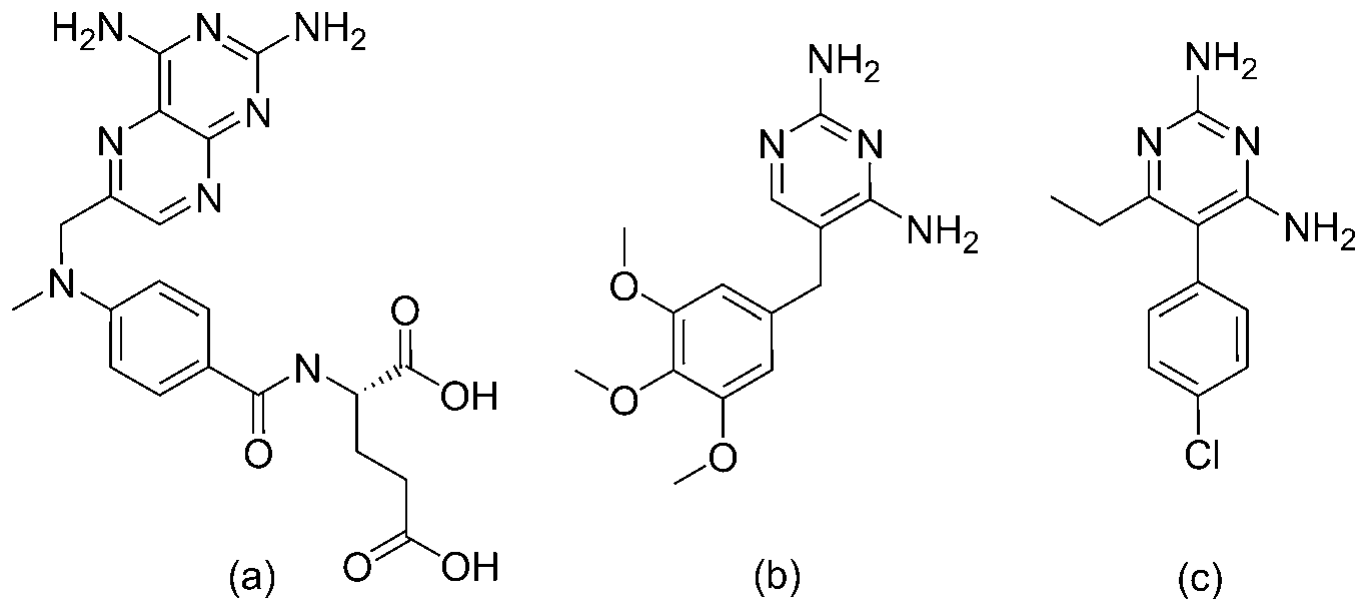
Known DHFR inhibitors: methotrexate (a); trimethoprim (b); pyrimethamine (c).

Folate metabolism has been successfully used as a drug target in a number of diseases such as cancer, bacterial infections and malaria. In particular, the enzyme dihydrofolate reductase (DHFR) is a clinically validated drug target in some diseases.^3^, ^4^ Figure 1 shows examples of known DHFR inhibitors. Although folate metabolism is a potential drug target in *Trypanosoma* and *Leishmania*,^5^ known DHFR inhibitors are not potent inhibitors of parasite growth. One possible explanation involves pteridine reductase 1 (PTR1), an NADPH-dependent enzyme that not only carries out the reduction of biopterin to dihydrobiopterin and dihydrobiopterin to tetrahydrobiopterin, but also the reduction of dihydrofolate to tetrahydrofolate.^6^, ^7^ Thus, PTR1 serves as a possible by-pass/resistance mechanism in *Leishmania* to DHFR inhibitors.^6^–^8^ Data from our laboratory, however, indicates that PTR1 may be a drug target in its own right in *T. brucei*, as PTR1 knockdown by RNA interference in the bloodstream form of *T. brucei* results in loss of viability in culture and loss of virulence in animal models of infection.^9^ These observations are strongly indicative that *Tb*PTR1 is a promising drug target alone or in combination with DHFR inhibitors for developing a novel treatment for HAT. However, for PTR1 to be considered a truly viable drug target, further chemical evidence of essentiality and druggability is required.

**Figure 2:**
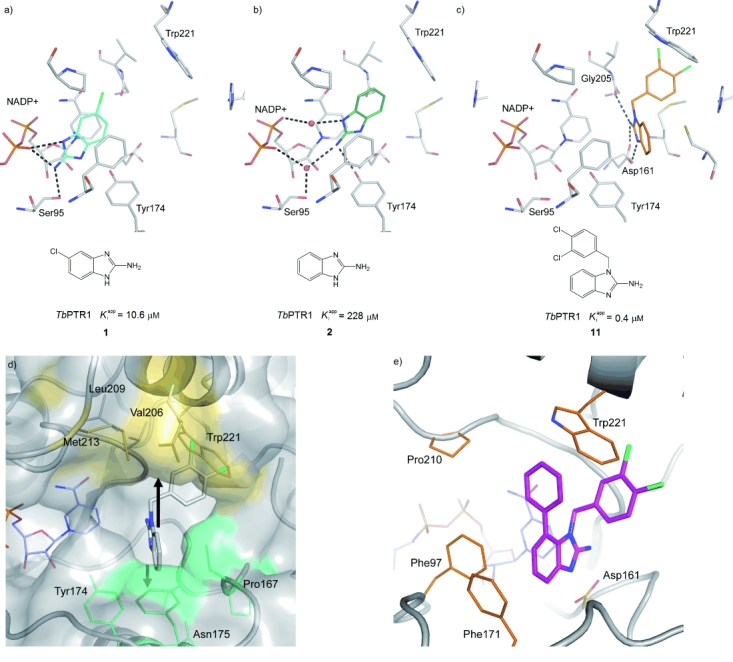
Depending on the substitution pattern, the aminobenzimidazole scaffold adopts three different binding modes. Crystal structures of the inhibitors are shown as follows:^13^ a) With only a small substitution in the 6-position, the inhibitor forms direct hydrogen bonds with the phosphate group of the co-factor NADP^+^. b) The unsubstituted scaffold forms water-mediated hydrogen bonds (red spheres) with the phosphate group of NADP^+^. c) When the scaffold is substituted at N1, the compound binds in an area of the binding site that is perpendicular to the canonical binding site.^13^ d) Hydrophobic pockets accessible by substitution of compound **11.** The surfaces of the pockets for substitutions at the 7- and 4-positions of **11** are coloured yellow and green, respectively. e) X-ray crystal structure of compound **32** (shown in magenta) showing use of the 7’ hydrophobic pocket and the edge–face interaction of the 7-phenyl group with Trp221.

Known inhibitors of PTR1 are based on diaminopteridine (methotrexate) and diaminopyrimidine (trimethoprim and pyrimethamine) templates (Figure 1).^10^, ^11^ Whilst these templates have a very strong binding interaction with the enzyme, they also have a high polar surface area (PSA), which may cause problems with blood–brain barrier (BBB) permeability and is associated with solubility problems. In addition, most of the known PTR1 inhibitors are not particularly selective and could also inhibit human DHFR, causing toxicity issues.^10^, ^12^ Therefore, we recently reported the virtual screening of a fragment library to identify new scaffolds with a lower PSA.^13^ The key compound series identified from this process was based on the aminobenzimidazole scaffold (Figure 2). X-ray crystallography demonstrated that this scaffold bound in three different binding modes, depending on the substituents; two of these were in the pterin binding site (Figure 2a and b, compounds **1** and **2**, respectively), whilst in the other binding mode, the ligand occupied an area of the active site perpendicular to the canonical binding site (Figure 2c, compound **11**). This part of the PTR1 binding site possesses pronounced differences compared to the DHFR active site and thus selectivity over DHFR was readily achieved. Two hydrophobic pockets are located close to the benzimidazole moiety in the canonical binding site, and can be explored for compound optimisation (Figure 2d). Substituting the 7-position of **11** led to **32** (Figure 2e) with an inhibition constant in the low nanomolar range (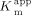
=7 nm). Despite this potency and favourable physicochemical properties, no activity against trypanosomes in vitro was observed. The reasons for this remain unclear.

Herein, we discuss in detail the medicinal chemistry programme around the aminobenzimidazole derivative adopting the alternative binding mode and structure–activity relationships (SAR) of the series. In addition, we discuss the antiparasitic activity of these compounds and the implications for chemical validation of the target.

## Results

### Initial hit exploration

**Figure 3:**
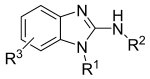
Modifications made to the aminobenzimidazole template to explore structure–activity relationships.

Initial work concerned systematic SAR studies around compound **11**, guided by structure-based design, crystallographically determined binding modes and activity data. The key substitutions made are shown in Figure 3.

#### N1 and N2 substituents

**Scheme 1:**
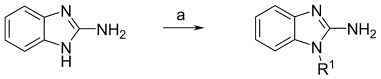
Synthetic route to N1-substituted 2-aminobenzimidazoles. *Reagents and conditions*: a) KOH, EtOH, R^1^X, 20 °C, 18 h, 47–88 %.

Compounds were either purchased or synthesised using the general method outlined in Scheme 1. Compounds were made by treating the 2-aminobenzimidazole under mild basic conditions with the appropriate alkylating agent. Reaction conditions were optimal at room temperature, where the reactions were generally clean with substitution occurring exclusively on the N1 nitrogen. Recovered product yields were in the range of 47 to 88 % of the theoretical maximum. In this way analogues were obtained to explore SAR around the N1-position. Key SAR data are shown in Table 1.

**Table 1:** Activity of N1- and N2-substituted compounds against *Tb*PTR1

Compd	R^1^ group	R^2^ group	*Tb*PTR1 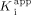 [μm][a]
**2**[b]	H	H	288
**3**[b]	Et	H	>200
**4**	C(=O)Ph	H	>60
**5**[b]	CH_2_CH_2_Ph	H	24
**6**	CH_2_Ph	H	16
**7**	CH_2_-(2-chlorobenzene)	H	10
**8**	CH_2_-(3-chlorobenzene)	H	7.5
**9**	CH_2_-(4-chlorobenzene)	H	5.4
**10**	CH_2_-(2,5-dichlorobenzene)	H	3.7
**11**[b]	CH_2_-(3,4-dichlorobenzene)	H	0.4
**12**	CH_2_-(3,5-dichlorobenzene)	H	8.8
**13**	CH_2_-(2-methylbenzene)	H	15
**14**	CH_2_-(2-fluorobenzene)	H	21
**15**	CH_2_-(4-methylbenzene)	H	15
**16**	CH_2_-(4-*tert*-butylbenzene)	H	4.2
**17**	CH_2_-(3-trifluoromethylbenzene)	H	6.3
**18**	CH_2_-(4-bromobenzene)	H	3.1
**19**	CH_2_-pyrid-2-yl	H	53
**20**	CH_2_-pyrid-3-yl	H	54
**21**	CH_2_-naphth-1-yl	H	6.1
**22**	CH_2_-naphth-2-yl	H	2.8
**23**	CH_2_-(3,4-dichlorobenzene)	C(=O)CH_3_	>60

[a] Data for each compound was determined in duplicate. 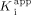 values were calculated using BatchKi software (BioKin); see Shanks et al. for more details.^14^

[b] Data for these compounds have been reported previously.^13^

Compounds **3** and **4**, where the N1 nitrogen is substituted with ethyl and benzoyl groups respectively, were inactive. However, compounds with an aromatic substituent attached to a flexible linker showed inhibition. A one-carbon spacer (compound **6**) appeared to give more potent inhibition than a two-carbon spacer (compound **5**); phenyl, pyridine and naphthyl derivatives appeared tolerated (compounds **6**, **19**–**22**); lipophilic and chlorine substituents on the phenyl ring (compounds **7**–**18**) gave a distinct SAR but none of the compounds were more potent than or at least equipotent to compound **11**. When N2 of **11** was acetylated, the compound was essentially inactive (compound **23** in Table 1).

#### R^3^ substituent

**Scheme 2:**
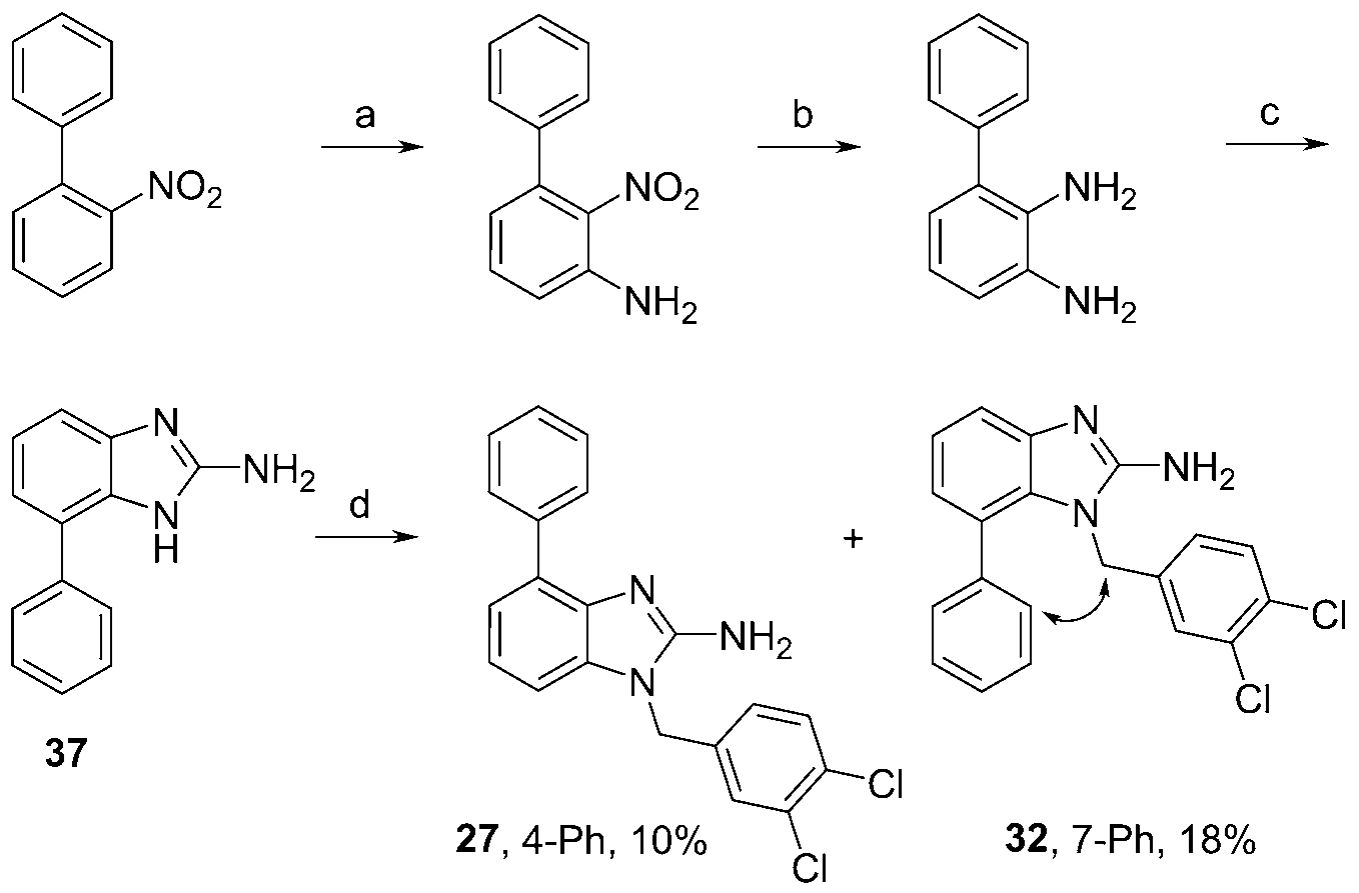
Synthetic route to compounds **27** and **32**. Optimisation of R^3^. The NOESY interaction between the *ortho* proton of the phenyl ring and the N1-CH_2_ group in **32** is indicated. *Reagents and conditions*: a) NH_2_OMe, *t*BuOK, CuCl, DMF, 1 h RT, 20 % (as in References 19, 20); b) EtOH, Sn(II)Cl_2_, 140 °C, MW, 10 min, >97 %; c) BrCN in MeCN, EtOH 70 °C, 1.5 h, 63 %; d) 3,4-dichlorobenzylbromide, EtOH, KOH, 20 °C, 18 h.

Preliminary optimisation of the N1-position revealed that the 3,4-dichlorobenzyl substituent (compound **11**) was the most favourable group in that position. Based on the crystallographically determined binding mode of **11** (Figure 2c), modelling predicted further enhancements in activity could be achieved in particular by substituents on the 4- and 7-positions of the aminobenzimidazole filling hydrophobic pockets close to the scaffold (Figure 2d).^13^ The general synthetic route employed, shown in Scheme 2, relies on the alkylation of the benzimidazole N1 in the final step, affording mixtures of 4- and 7- substituted benzimidazoles, which were then separated by chromatographic methods. Scheme 2 shows the route employed to make the 4- and 7-phenyl benzimidazole analogues. The 4’ and 7’ alkoxy analogues were synthesised from corresponding alkylation of the commercially available 2-amino-3-nitrophenol,^15^, ^16^ reduction of the nitro group^17^ and cyclisation.^18^ Other synthetic methods were attempted, which would selectively give access to either the 4- or 7-substituted products, but these methods either failed or were lower yielding than producing the final product mixtures and separating these by chromatography. The absolute regiochemistry of the isolated final compounds was assigned on the basis of the ^1^H NMR spectra, the NOESY spectra, and X-ray crystallography, where we have reported a complex with the enzyme.^13^ In the NOESY spectra for **31** and **32**, we were able to see an interaction between the protons on the 7-substituent with the N1-CH_2_-benzyl protons as indicated in Scheme 2.

Biological data for compounds with substituents in the 4- or 7-positions is shown in Table 2. All compounds with substituents in the 4- or 7-position are selective for *Tb*PTR1 over human and *Tb*DHFR. Small substituents in the 4- or 7-position (compounds **24**, **28**, **29** and **33**) did not lead to a significant improvement in potency against *Tb*PTR1 compared to **11**. The slightly larger propoxy substituent in the 4-position (compound **25**) gave similar potency to the parent compound **11**, whereas the same substituent in the 7-position (compound **30**) increased potency by almost 15-fold. The even more bulky and rigid phenyl and *O*-benzyl groups decreased potency in the 4-postion (**26** and **27**) while showing increased potency in the 7-position (**31** and **32**) affording the most potent compound in this series (compound **32**), which has a 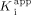 value of 7 nm.

**Table 2:** Activity of R^3^-substituted compounds against *Tb*PTR1, *Tb*DHFR and human DHFR, and *T. brucei* and MRC5 cells. R^1^ in all compounds is 3,4-dichlorobenzyl

Compd	R^3^	*Tb*PTR1 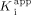 [μm]	*Hs*DHFR IC_50_ [μm]	*Tb*DHFR IC_50_ [μm]	*T. brucei*[a] EC_50_ [μm]	MRC5 EC_50_ [μm]
**11**[b]	H	0.8	>50	>50	11	27
**24**	4-Cl	2.3	>50	>30	ND[d]	ND
**25**	4-O(CH_2_)_2_CH_3_	0.31	>50	>30	ND	ND
**26**	4-OBn	16	>50	>50	>30	>30
**27**	4-Ph	>60	>30	>30	ND	ND
**28**[c]	4-OMe	0.46	>50	>30	ND	ND
**29**[b]	7-Cl	0.51	>50	>30	ND	ND
**30**[b]	7-O(CH_2_)_2_CH_3_	0.047	>50	>30	9.6	21
**31**	7-OBn	0.098	>50	>50	6.7	17
**32**[b]	7-Ph	0.007	>50	>50	9.9	>30
**33**	7-OMe	0.65	>50	>30	ND	ND
**34**	5,6-dimethyl	3.9	>30	>30	ND	ND

[a] In vitro cell assay conditions used were as reported previously.^21^

[b] Some data for these compounds have been reported previously.^13^

[c] Contaminated with ∼25 % of compound **33**.

[d] ND=Not determined.

#### Parasite activity of PTR1 inhibitors

Unfortunately, cell efficacy did not track the PTR1 enzyme potency (Table 2) and no significant improvements in efficacy against *T. brucei* were observed going from compound **11** to compound **32**. In addition, some toxicity was evident with some compounds against the mammalian MRC5 cell line in the micromolar range. Mammalian cells do not possess PTR1, so off-target effects may be involved. Inhibition of mammalian DHFR can be discounted, since none of these compounds inhibited this enzyme. Possible reasons for the poor trypanocidal activity were investigated.

**Figure 4:**
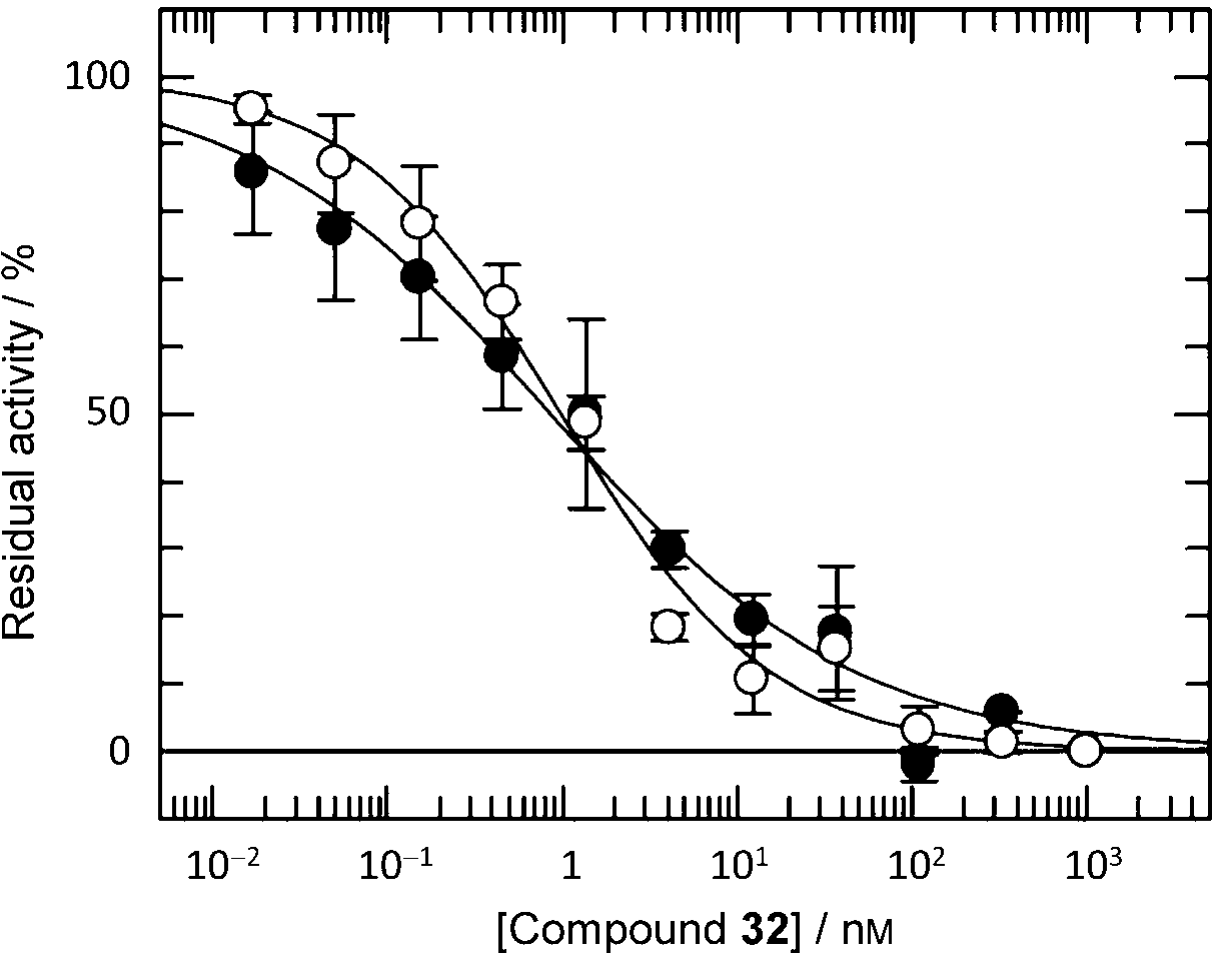
Activity of compound **32** against recombinant PTR1 and endogenous PTR1 in a trypanosome lysate: recombinant PTR1 (○); clarified trypanosome lysate (•). The uninhibited rates were 2.5 and 1.7 pmol min^−1^ mL^−1^, respectively.

To determine whether there were any differences between recombinant and endogenous PTR1, the potency of **32** was measured in clarified lysates of *T. brucei* using a specific HPLC-based assay instead of the cytochrome *c*-coupled assay that is only suitable for studies on the purified enzyme (Figure 4). Adequate extraction of the parasites was confirmed when trypanothione reductase activity in the lysates (63.3±1.7 nmol min^−1^ mg^−1^) was found to be in good agreement with previously published data.^22^ The potency of compound **32** was found to be virtually identical against PTR1 in cell lysates and the purified recombinant enzyme (IC_50_=0.88±0.13 nm, Hill slope=0.5, and 0.99±0.13 nm, Hill slope 0.73, respectively). However, due to the shallower Hill slope in the cell extract, higher concentrations of inhibitor are required to completely inhibit PTR1 activity (∼1 μm). The low Hill slopes may be due to PTR1 being a tetramer, and there being negative cooperativity on binding to the individual subunits.

In order to achieve activity against the parasite, these inhibitors need to pass through the cell membrane to reach PTR1 in the cytosol. In general, the compounds have reasonable properties for cellular penetration at physiological pH: molecular weights less than 400, one hydrogen-bond acceptor and two or three hydrogen-bond donors. The calculated log *D* values are in the range of 3.5 to 4.5, and the experimental values are in the range of 3.0 to >4 (Table 3). Therefore, the compounds are within acceptable ranges for cellular penetration, albeit at the higher end of lipophilicity. However, given the high protein binding of these compounds, it is possible that only the free fraction (1–3 %) is able to reach the target in whole cells. This does not seem to be the case, since inclusion of either foetal calf serum (10 % *v*/*v*) or bovine serum albumin (1 mg mL^−1^) in the assay only increased the IC_50_ values against recombinant PTR1 by two- to threefold (2.2±0.4 and 2.9±0.1 nm, respectively).

**Table 3:** Physicochemical properties of compounds with trypanocidal activity

Compd	MW	log *D*		PSA	Solubility	Exptl PB	Predicted
		Calcd	Exptl	[Å^2^]	[μm]	[%]	BBB[b]
**11**	292	3.0	>4	44	200–300	97	+
**30**	350	3.6	3.0	53	ND	99	+
**31**	398	4.4	>4	53	ND	98	+
**32**	368	4.7	3.7	44	ND	99	+

[a] Calcd, calculated; Exptl, experimental (measured); PSA, polar surface area; PB, protein binding.

[b] Blood–brain barrier (BBB) permeability predicted by StarDrop (http://www.optibrium.com).

## Discussion

During this work, we successfully prepared a number of 2-aminobenzimidazoles as new PTR1 inhibitors. This work was guided using structure-based design and medicinal chemistry principles. The SAR that we observe here can be explained by the crystal structure information previously reported on complexes with the enzyme.^13^

### Binding modes

All of the compounds that are reported here have a substituent at the 1-position (R^1^). Consequently, binding in modes similar to one or two (Figure 2a and 2b) is highly unlikely, as this would disrupt the hydrogen-bonding interactions from N1. We therefore assume that these compounds bind in a similar manner as compound **11** in binding mode three (Figure 2c). In this binding mode, the N1 undergoes no hydrogen-bonding interaction with the protein, so substituents on this position should not disrupt hydrogen-bonding interactions. However, compound **3** with an ethyl substituent, failed to give significant binding; whilst the ethyl substituent probably did not disrupt binding, it did not give any significant interactions with the enzyme, especially with Trp221, which is known to contribute substantially to binding affinity (unpublished results derived from other compound series). The benzoate substituent of compound **4** also diminished binding affinity. This is most likely due to a decrease of the p*K*_a_ of the N3 nitrogen of the aminobenzimidazole, thereby effecting the hydrogen-bonding interaction with Asp161 and to the change in geometry of the substituent caused by the carbonyl group, which does not allow the compound to form favourable interactions with Trp221.

As was previously reported for compound **11**,^13^ the crystal structure in complex with PTR1 revealed that the chloro-substituents on positions 3 and 4 fill a hydrophobic pocket formed by Val206, Trp221, Lys224 and Leu263 (Figure 2c). Removing or replacing one or more of the chlorine atoms and/or changing the substitution pattern (compounds **6**–**10**, **12**–**18**) resulted in weaker inhibitors, probably due to less than optimal filling of this hydrophobic pocket. Similarly, the pyridyl analogues (**19** and **20**) are weaker inhibitors, probably due to the lack of hydrogen-bonding partners for the pyridyl nitrogen atoms in the hydrophobic pocket and due to the pyridyl substituents not filling the hydrophobic pocket as optimally. The naphthyl derivates (**21** and **22**) were designed to mimic the 3,4-dichlorophenyl group of **11**. Compound **22** is only a slightly weaker (fourfold) inhibitor than compound **11**, whilst compound **21** is tenfold weaker. This may be due to 2-naphthyl derivative occupying the hydrophobic cavity in a slightly more favourable conformation than compound **21**.

The acetate derivative **23** was synthesized in order to probe the flexibility of the binding pocket around Gly205 and Asp161, which form hydrogen bonds with the amino group of compound **11** in the binding mode determined by crystallography.^13^ There was a complete loss of activity of compound **23**, indicating that the binding site is rather rigid in that area and does not accommodate substituents of the amino group.

Our analysis of the crystal structure of PTR1⋅**11** had revealed two hydrophobic pockets that could be reached by substituents from the 4- and 7-positions (Figure 2d).^13^ The pocket in the 7-position bounded by residues Trp221, Met213, Val206 and Leu209 appeared to be larger than the pocket in the 4-position, formed by residues Pro167, Tyr174 and Asn175. Indeed, smaller substituents at the 4- or 7-position are tolerated in both pockets (compounds **24**, **28**, **29** and **33**) but do not lead to a significant increase in binding affinity. The slightly larger propoxy group of compounds **25** and **30** leads to similar activity against the PTR1 compared to **11** when attached in the 4-position, but to an almost 20-fold increase when attached in the 7-position. A phenyl group in the 4-position (compound **27**) leads to a greater than tenfold decrease in potency compared to compound **11**, probably caused by steric clashes in this rather small hydrophobic pocket. In contrast, attachment of the same group to the 7-position results in an almost 100-fold increase in potency, probably due to edge–face interactions of this group with Trp221 and displacement of water molecules from the hydrophobic pocket (Figure 2e). In general, no enhancement in activity was achieved by accessing the putative pocket at the 4-position.

### Parasite activity and chemical validation

Several potential reasons for the disappointingly large discrepancy between PTR1 enzyme potency and cell efficacy have been investigated using compound **32**:

• *Physicochemical factors*: The physicochemical properties of all the trypanocidal compounds appear within acceptable ranges for cellular penetration, albeit at the higher end of lipophilicity (Table 3). However, the possibility that compound **32** is effectively sequestered in cellular lipids and therefore unable to reach its cytosolic target enzyme or is actively effluxed out of the cells cannot be definitively excluded by our current studies.

• *Differences between the recombinant and endogenous enzyme*: It is clear that differences between recombinant and endogenous native PTR1 do not appear to play a significant role. Interestingly, the consistently low Hill slope observed here and previously^13^ could indicate negative cooperativity restricting ligand occupancy on all four subunits of the tetramer.^6^

• *Extent of enzyme inhibition*: Although the extent to which PTR1 has to be inhibited to cause growth effects is not known with certainty, gene knockout experiments indicate that halving enzyme activity has no effect on growth and RNAi depletion studies suggest >90 % knockdown of PTR1 protein is required to exert a trypanocidal effect.^9^ Therefore, it is possible that compounds have insufficient potency at the enzyme level to cause an effect at the cellular level. PTR1 has an apparent *K*_m_ (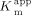) value of 167±54 nm using the cytochrome *c*-coupled assay^14^ used to determine the 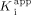 for **32**, and the crystal structure of **32** indicates that this inhibitor is competitive with respect to the substrate dihydrobiopterin (Figure 2e). Thus, the inhibition constant (*K*_i_) for **32** can be calculated from Equation (1) for competitive inhibition.


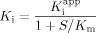
(1)

Applying Equation (1) yields a *K*_i_ value of 2.3 nm under our assay conditions, where the substrate concentration (*S*) is 350 nm. Using the direct HPLC method, the 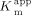 value for PTR1 is 25 nm,^14^ which is sevenfold lower than the value obtained in the cytochrome *c* assay. This is due to the latter assay generating quinonoid dihydrobiopterin, which can then rearrange to form 7,8-dihydrobiopterin.^23^ Subsequent work from this laboratory has established that *T. brucei* PTR1 can also reduce quinonoid dihydrobiopterin to tetrahydrobiopterin (Ong and Fairlamb, unpublished). Thus, the 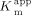 value determined by the cytochrome *c* method is a hybrid 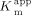 value for a mixture of these substrates. Additional studies from our laboratory indicate that the total intracellular biopterin concentration in *T. brucei* is 480 nm of which 98 % is present in the tetrahydro form (Ong and Fairlamb, unpublished results). Assuming that all of this has to be oxidised to dihydrobiopterin for lethality and that enzyme inhibition must be maintained at 90 % of normal levels to successfully deplete the tetrahydrobiopterin levels, then the required free concentration of **32** can be calculated from Equation (2).


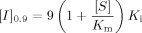
(2)

Using these assumptions (*S*=480 nm, *K*_m_=25 nm, *K*_i_=2.3 nm), the predicted concentration of unbound **32** required to maintain 90 % inhibition would be 418 nm, consistent with the results in Figure 4. A similar calculation for 95 % inhibition yields 883 nm. Combined with the two- to threefold decrease in potency due to protein binding, a trypanocidal effect would only be expected in the 1–2 μm range, which is reasonably consistent with the observed EC_50_ value of 10 μm in Table 2. These theoretical calculations provide a plausible explanation for the 1000-fold decrease in potency between target and cell, and underline the need for the development of substantially more potent competitive inhibitors since the current ratio of *K*_m_/*K*_i_ (25 and 2.3 nm, respectively) is only 10.

From Equation (2), [*I*]_0.9_ is inversely related to *K*_m_/*K*_i_. In sharp contrast, the *K*_m_/*K*_i_ ratio for the folate analogue methotrexate against murine DHFR is 260 000^24^ and 30 000 for human DHFR.^25^ In trypanosomes, the only known targets for methotrexate are DHFR and PTR1 with *K*_i_ values of 0.15 and 3.6 nm, respectively.^14^ Significantly, methotrexate, which is equipotent with **32** in respect of *Tb*PTR1 inhibition, displays similar cell potency in a genetically engineered cell line lacking DHFR compared to the parental cell line used here (EC_50_ values of 17.9 and 9.9 μm, respectively). This supports the idea that the poor cellular potency has more to do with *K*_m_/*K*_i_ ratios than the specific physicochemical explanations indicated for this particular novel series.

## Conclusions

We have established SAR for a series of novel PTR1 inhibitors. The most potent compounds of this series have appropriate druglike properties and are highly selective (>7 000-fold) for PTR1 over human or trypanosomal DHFR. Compounds **32** and **30** are the most potent and selective *Tb*PTR1 inhibitors disclosed in the literature to date and will hopefully prove to be useful pharmacological tools for the exploration of the role PTR1 plays in the survival and growth of these parasites. However, in order to produce effective drug candidates directed solely at PTR1, potency will need to be enhanced by at least another two orders of magnitude.

## Experimental Section

The chemistry and biology experimental sections are in the Supporting Information.

## References

[1] Pink R, Hudson A, Mouries MA, Bendig M (2005). Nat. Rev. Drug Discov..

[2] Fairlamb AH (2003). Trends Parasitol..

[3] Gangjee A, Jain HD, Kurup S (2007). Anti-Cancer Agents Med. Chem..

[4] Gangjee A, Jain HD, Kurup S (2008). Anti-Cancer Agents Med. Chem..

[5] Berriman M, Ghedin E, Hertz-Fowler C, Blandin G, Renauld H, Bartholomeu DC, Lennard NJ, Caler E, Hamlin NE, Haas B, Bohme W, Hannick L, Aslett MA, Shallom J, Marcello L, Hou LH, Wickstead B, Alsmark UCM, Arrowsmith C, Atkin RJ, Barron AJ, Bringaud F, Brooks K, Carrington M, Cherevach I, Chillingworth TJ, Churcher C, Clark LN, Corton CH, Cronin A, Davies RM, Doggett J, Djikeng A, Feldblyum T, Field MC, Fraser A, Goodhead I, Hance Z, Harper D, Harris BR, Hauser H, Hostetter J, Ivens A, Jagels K, Johnson D, Johnson J, Jones K, Kerhornou AX, Koo H, Larke N, Landfear S, Larkin C, Leech V, Line A, Lord A, MacLeod A, Mooney PJ, Moule S, Martin DMA, Morgan GW, Mungall K, Norbertczak H, Ormond D, Pai G, Peacock CS, Peterson J, Quail MA, Rabbinowitsch E, Rajandream MA, Reitter C, Salzberg SL, Sanders M, Schobel S, Sharp S, Simmonds M, Simpson AJ, Talton L, Turner CMR, Tait A, Tivey AR, Van Aken S, Walker D, Wanless D, Wang SL, White B, White O, Whitehead S, Woodward J, Wortman J, Adams MD, Embley TM, Gull K, Ullu E, Barry JD, Fairlamb AH, Opperdoes F, Barret BG, Donelson JE, Hall N, Fraser CM, Melville SE, El-Sayed NM (2005). Science.

[6] Nare B, Hardy LW, Beverley SM (1997). J. Biol. Chem..

[7] Bello AR, Nare B, Freedman D, Hardy L, Beverley SM (1994). Proc. Natl. Acad. Sci. USA.

[8] Cunningham ML, Titus RG, Turco SJ, Beverley SM (2001). Science.

[9] Sienkiewicz N, Ong HB, Fairlamb AH (2010). Mol. Microbiol..

[10] Cavazzuti A, Paglietti G, Hunter WN, Gamarro F, Piras S, Loriga M, Alleca S, Corona P, McLuskey K, Tulloch L, Gibellini F, Ferrari S, Costin MP (2008). Proc. Natl. Acad. Sci. USA.

[11] Hardy LW, Matthews W, Nare B, Beverley SM (1997). Exp. Parasitol..

[12] Tulloch LB, Martini VP, Iulek J, Huggan JK, Lee JH, Smith TK, Suckling CJ, Hunter WN (2010). J. Med. Chem..

[13] Mpamhanga CP, Spinks D, Tulloch LB, Shanks EJ, Robinson DA, Collie IT, Fairlamb AH, Wyatt PG, Frearson JA, Hunter WN, Gilbert IH, Brenk R (2009). J. Med. Chem..

[14] Shanks EJ, Ong HB, Robinson DA, Thompson S, Sienkiewicz N, Fairlamb AH, Frearson JA (2010). Anal. Biochem..

[15] Doherty EM, Fotsch C, Bannon AW, Bo YX, Chen N, Dominguez C, Falsey J, Gavva NR, Katon J, Nixey T, Ognyanov VI, Pettus L, Rzasa RM, Stec M, Surapaneni S, Tamir R, Zhu JW, Treanor JJS, Norman MH (2007). J. Med. Chem..

[16] Zhang L, Fan JH, Vu K, Hong K, Le Brazidec JY, Shi JD, Biamonte M, Busch DJ, Lough RE, Grecko R, Ran YQ, Sensintaffar JL, Kamal A, Lundgren K, Burrows FJ, Mansfield R, Timony GA, Ulm EH, Kasibhatla SR, Boehm MF (2006). J. Med. Chem..

[17] Rangarajan M, Kim JS, Sim SP, Liu A, Liu LF, LaVoie EJ (2000). Bioorg. Med. Chem..

[18] Leonard NJ, Curtin DY, Beck KM (1947). J. Am. Chem. Soc..

[19] Seko S (1995).

[20] Seko S, Miyake K, Kawamura N (1999). J. Chem. Soc. Perkin Trans. 1.

[21] Spinks D, Shanks EJ, Cleghorn LAT, McElroy S, Jones D, James D, Fairlamb AH, Frearson JA, Wyatt PG, Gilbert IH (2009). ChemMedChem.

[22] Vickers TJ, Fairlamb AH (2004). J. Biol. Chem..

[23] Armarego WLF, Randles D, Taguchi H (1983). Eur. J. Biochem..

[24] Williams JW, Morrison JF, Duggleby RG (1979). Biochemistry.

[25] Appleman JR, Prendergast N, Delcamp TJ, Freisheim JH, Blakley RL (1988). J. Biol. Chem..

