# Supplementary material for: Design, Synthesis and Biological Evaluation of Novel Inhibitors of Trypanosoma brucei Pteridine Reductase 1
Source: ChemMedChem. 2010 Nov 25;6(2):302–8. doi: 10.1002/cmdc.201000450 (PMC3047710; doi:10.1002/cmdc.201000450)
Supplement: Supplementary file 1 [file cmdc0006-0302-SD1.pdf]

CHEMMEDCHEM
Chemistry & Drug Discovery

Supporting Information

© Copyright Wiley-VCH Verlag GmbH & Co. KGaA, 69451 Weinheim, 2011

Design, Synthesis and Biological Evaluation of Novel Inhibitors of *Trypanosoma brucei* Pteridine Reductase 1

Daniel Spinks, Han B. Ong, Chidochangu P. Mpamhanga, Emma J. Shanks,
David A. Robinson, Iain T. Collie, Kevin D. Read, Julie A. Frearson, Paul G. Wyatt, Ruth Brenk,
Alan H. Fairlamb, and Ian H. Gilbert*[a]

cmdc_201000450_sm_miscellaneous_information.pdf

Experimental section

Chemistry

^{1}H NMR spectra were recorded on either a Bruker Avance DPX 500 or on a Bruker Avance 300 spectrometer. Chemical shifts (δ) are expressed in ppm. Signal splitting patterns are described as singlet (s), broad singlet (bs), doublet (d), triplet (t), quartet (q), multiplet (m) or combinations thereof. LC-MS analyses were performed with either an Agilent HPLC 1100 series connected to a Bruker Daltonics MicrOTOF, or an Agilent Technologies 1200 series HPLC connected to an Agilent Technologies 6130 quadrupole LC/MS, both instruments were connected to an Agilent diode array detector. LCMS chromatographic separations were conducted with a Phenomenex Gemini C18 column, 50 × 3.0 mm, 5 µm particle size; mobile phase, water / acetonitrile + 0.1% HCOOH 80:20 to 5:95 over 3.5 min, and then held for 1.5 min; flow rate 0.5 ml min^{-1}. High resolution electrospray measurements were performed on a Bruker Daltonics MicrOTOF mass spectrometer. Thin layer chromatography (TLC) was carried out on Merck silica gel 60 F254 plates using UV light and / or $KMnO_4$ for visualization. TLC data are given as the Rf value with the corresponding eluant system specified in brackets. Column chromatography was performed using RediSep® 4 or 12 g silica prepacked columns. All reactions were carried out under dry and inert conditions unless otherwise stated.

Compounds **1** and **2** were obtained from Sigma-Aldrich, **3**, **13** from ChemDiv, **5** from Asinex, **16**, **17**, **18** from ChemBridge and **6**, **14**, **15** from Enamine. All these compounds were found to be >97% pure by LC-MS.

The synthesis of compounds 11,29, 30 &32 has been described previously.[1]

1-benzoyl-2-aminobenzimidazole (4)

2-Aminobenzimidazole (**2**) (1 g, 7.52 mmol) was taken into acetone (30 ml) and cooled to 0°C. Triethylamine (1.5 equiv. 1.58 ml, 11.3 mmol) was added, followed by the dropwise addition of benzoyl chloride (1.16 g, 8.27 mmol). The reaction was taken out of the ice/water bath and stirred at 20°C for 18 h. The solution was then concentrated by removing the acetone, diluted with 40 ml dichloromethane, and washed with sodium bicarbonate solution (1x20 ml), water (1x30 ml) and brine

(2x20 ml). The organic layer was dried with magnesium sulphate and concentrated to remove the dichloromethane to afford compound **4** as a white solid (1.63 g, 91%). ^{1}H NMR and LCMS confirmed purity at >98%. ^{1}H NMR (300 MHz, DMSO): δ = 6.02 1H, d J=8.0Hz, ArH), 6.67 (1H, m, ArH), 7.05 (1H, dt J=7.8, 1.0 Hz), ArH, 7.18 (1H, m, ArH), 7.19 (2H, bs, NH_2), 7.61 (2H, m, 2ArH), 7.72 (3H, m, ArH). LCMS (ES^+): m/z (%) 238 $(M+H)^+$ retention time 1.4 min. HRMS (ES^+): calcd for $C_{14}H_{12}N_3O$ $[M+H]^+$ 238.0975, found 238.0963.

1-(2-Chlorobenzyl)-1H-benzo[d]imidazol-2-amine (7)[2]

2-Aminobenzimidazole (**2**) (500 mg, 3.76 mmol), potassium hydroxide flakes (317 mg, 5.64 mmol) and 2-chlorobenzyl bromide (634 mg, 3.95 mmol) were stirred in 15 ml ethanol at 20°C for 18 h. The solution was then concentrated by removing the ethanol and diluted with 40 ml ethylacetate, and washed with sodium bicarbonate solution (1x20 ml), water (1x30 ml) and brine (2x20 ml). The organic layer was dried with magnesium sulphate and concentrated to remove the ethyl acetate. The crude product was purified by flash column chromatography (6% methanol/ dichloromethane eluant), to yield compound **7** as a white solid (850 mg, 88%). ^{1}H NMR and LCMS confirmed purity at >98%. ^{1}H NMR (500 MHz, DMSO): δ = 5.32 (2H, s, CH_2), 6.50 (1H, dd J=7.7, 1.3 Hz), ArH), 6.58 (2H, bs, NH_2), 6.80 (1H, dt J=7.9, 1.0 Hz, ArH), 6.89 (1H, d J=7.4Hz, ArH), 6.95 (1H, m, ArH), 7.18 (1H, d J=7.7Hz, ArH), 7.22 (1H, dt J=7.6, 1.2Hz, ArH), 7.31 (1H, dt J=7.8, 1.6Hz, ArH), 7.53 (1H, dd J=7..9, 1.1Hz,, ArH). LCMS (ES^+): m/z (%) 258 $(M+H)^+$ retention time 0.8 min. HRMS (ES^+): calcd for (Cl^{35}) $C_{14}H_{13}ClN_3$ [M+H]+ 258.0793, found 258.0795.

1-(3-Chlorobenzyl)-1H-benzo[d]imidazol-2-amine (8)[2, 3]

Prepared as for compound **7**, starting from 2-aminobenzimidazole (**2**) (500 mg, 3.76 mmol), potassium hydroxide flakes (317 mg, 5.64 mmol) and 3-chlorobenzyl bromide (634 mg, 3.95 mmol) in 15 ml ethanol. The reaction was stirred at 20°C for 18 h and concentrated, washed, then concentrated again. The crude product was purified by flash column chromatography (6% methanol / dichloromethane eluant), to yield compound **8** as a white solid (650 mg, 59%). ^{1}H NMR and LCMS confirmed purity at >98%. ^{1}H NMR (500 MHz, DMSO): δ = 5.26 (2H, s, CH_2), 6.56 (2H, s, NH_2), 6.84 (1H, dt J=7.6, 1.1Hz), ArH), 6.94 (1H, dt J=7.6, 1.1Hz), ArH), 7.08 (1H, d J=7.5Hz, ArH), 7.14 (2H, m, 2ArH), 7.22

(1H, m, ArH), 7.34 (2H, m, 2ArH). LCMS (ES^+): m/z (%) 258 $(M+H)^+$ retention time 0.7 min. HRMS (ES^+): calcd for (Cl^{35}) $C_{14}H_{13}ClN_3$ [M+H]+ 258.0793, found 258.0789.

1-(4-Chlorobenzyl)-1H-benzo[d]imidazol-2-amine (9)[2, 4, 5]

Prepared as for compound **7**, starting from 2-aminobenzimidazole (**2**) (500 mg, 3.76 mmol), potassium hydroxide flakes (317 mg, 5.64 mmol) and 4-chlorobenzyl bromide (634 mg, 3.95 mmol) in 15 ml ethanol. The reaction was stirred at 20°C for 18 h and concentrated, washed, then concentrated again. The crude product was purified by flash column chromatography (6% methanol / dichloromethane eluant), to yield compound **9** as a white solid (740 mg, 67%). H NMR and LCMS confirmed purity at >98%. ^{1}H NMR (500 MHz, DMSO): δ = 5.26 (2H, s, CH_2), 6.57 (2H, s, NH_2), 6.83 (1H, dt J=7.6, 1.1Hz), ArH), 6.92 (1H, dt J=7.6, 1.1Hz), ArH), 7.06 (1H, d J=7.6Hz, ArH), 7.14 (1H, d J=7.7Hz, ArH), 7.23 (2H, bd J=8.5Hz, 2ArH), 7.40 (2H, bd J=8.5Hz, 2ArH). LCMS (ES^+): m/z (%) 258 $(M+H)^+$ retention time 0.8 min. HRMS (ES^+): calcd for (Cl^{35}) $C_{14}H_{13}ClN_3$ [M+H]+ 258.0793, found 258.0790.

1-(2,5-Dichlorobenzyl)-1H-benzo[d]imidazol-2-amine (10)

Prepared as for compound **7**, starting from 2-aminobenzimidazole (**2**) (500 mg, 3.76 mmol), potassium hydroxide flakes (317 mg, 5.64 mmol) and 2,5-dichlorobenzyl chloride (772 mg, 3.95 mmol) in 15 ml ethanol. The reaction was stirred at 20°C for 18 h and concentrated, washed, then concentrated again. The crude product was purified by flash column chromatography (6% methanol / dichloromethane eluant), to yield compound **10** as a white solid (650 mg, 59%). ^{1}H NMR and LCMS confirmed purity at >98%.

^{1}H NMR (500 MHz, DMSO): δ = 5.31 (2H, s, CH_2), 6.42 (1H, d J=2.5Hz, ArH), 6.60 (2H, s, NH_2), 6.82 (1H, dt , J=7.6, 1.1Hz, ArH), 6.97 (2H, m, 2ArH), 7.20 (1H, d J=7.6Hz, CH), 7.41 (1H, dd J=8.5, 2.6Hz, ArH), 7.59 (1H, d J=8.5Hz, ArH). LCMS (ES^+): m/z (%) 293 $(M+H)^+$ retention time 0.9 min. HRMS (ES^+): calcd for (2xCl^{35}) $C_{14}H_{12}Cl_2N_3$ [M+H]+ 292.0403, found 292.0391.

1-(3,4-Dichlorobenzyl)-1H-benzo(d)imidazol-2-amine (11)[6]

Prepared as for compound **7**, starting from 2-aminobenzimidazole (**2**) (500 mg, 3.76 mmol), potassium hydroxide flakes (317 mg, 5.64 mmol) and 3,4-dichlorobenzyl chloride (772 mg, 3.95 mmol) in 15 ml

ethanol. The reaction was stirred at 20°C for 18 h and concentrated, washed, then concentrated again to afford a cream coloured solid. The crude product was purified by flash column chromatography (6% methanol / dichloromethane eluant), to yield compound **11** as a white solid (512 mg, 47%). ^{1}H NMR and LCMS confirmed purity at >98%. ^{1}H NMR (500 MHz, DMSO): δ = 5.24 (2H, s, CH_2), 6.58 (2H, s, NH_2), 6.85 (H, dt J=7.6, 0.8Hz, ArH), 6.95 (1H, dt , J=7.6, 1.0Hz, ArH), 7.12 (3H, m, 3ArH), 7.47 (1H, d J=2.0Hz, ArH), 7.61 (1H, d J=8.3Hz, ArH). LCMS (ES^+): m/z (%) 293 $(M+H)^+$ retention time 0.8 min. HRMS (ES^+): calcd for ($2xCl^{35}$) $C_{14}H_{12}Cl_2N_3$ [M+H]+ 292.0403, found 292.0407.

1-(3,5-Dichlorobenzyl)-1H-benzo[d]imidazol-2-amine (12)

Prepared as for compound **7**, starting from 2-aminobenzimidazole (**2**) (500 mg, 3.76 mmol), potassium hydroxide flakes (317 mg, 5.64 mmol) and 3,5-dichlorobenzyl chloride (772 mg, 3.95 mmol) in 15 ml ethanol The reaction was stirred at 20°C for 18 h and concentrated, washed, then concentrated again. The crude product was purified by flash column chromatography (6% methanol / dichloromethane eluant), to yield compound **12** as a white solid (700 mg, 64%). ^{1}H NMR and LCMS confirmed purity at >98%. ^{1}H NMR (500 MHz, DMSO): δ = 5.27 (2H, s, CH_2), 6.60 (2H, s, NH_2), 6.86 (1H, dt , J=7.6, 1.1Hz, ArH), 6.95 (1H, dt , J=7.6, 1.0Hz, ArH), 7.14 (2H, dd J=18.5, 7.6Hz, 2ArH), 7.21 (2H, d J=1.9Hz, 2CH), 7.54 (1H, m, ArH). LCMS (ES^+): m/z (%) 293 $(M+H)^+$ retention time 0.9 min. HRMS (ES^+): calcd for ($2xCl^{35}$) $C_{14}H_{12}Cl_2N_3$ [M+H]+ 292.0403, found 292.0397.

1-(Pyridin-2-ylmethyl)-1H-benzo[d]imidazol-2-amine (19)

Prepared as for compound **7**, starting from 2-aminobenzimidazole (**2**) (500 mg, 3.76 mmol), potassium hydroxide flakes (317 mg, 5.64 mmol) and 2-(bromomethyl)pyridine hydrobromide salt (999 mg, 3.95 mmol) in 15 ml ethanol. The reaction was stirred at 20°C for 18 h and concentrated, washed, then concentrated again. The crude product was purified by flash column chromatography (10% methanol / dichloromethane eluant), to yield compound **19** as a white solid (615 mg, 73%). ^{1}H NMR and LCMS confirmed purity at >98%. ^{1}H NMR (500 MHz, DMSO): δ = 5.32 (2H, s, CH_2), 6.53 (2H, s, NH_2), 6.82 (1H, dt , J=7.6, 1.0Hz, ArH), 6.93 (1H, dt , J=7.6, 1.1Hz, ArH), 7.04 (2H, m, 2ArH), 7.15 (1H, d J=7.7Hz, ArH), 7.29 (1H, ddd J=7.5, 4.9, 0.9Hz, ArH), 7.75 (1H, dt J=7.7, 1.8Hz, ArH), 8.54 (1H, m, ArH). LCMS (ES^+): m/z (%) 225 $(M+H)^+$ retention time 0.6 min. HRMS (ES^+): calcd for $C_{13}H_{13}N_4$ [M+H]+ 225.1135, found 225.1133.

1-(Pyridin-3-ylmethyl)-1H-benzo[d]imidazol-2-amine (20)

Prepared as for compound **7**, starting from 2-aminobenzimidazole (**2**) (500 mg, 3.76 mmol), potassium hydroxide flakes (317 mg, 5.64 mmol) and 3-(bromomethyl)pyridine hydrobromide salt (999 mg, 3.95 mmol) in 15 ml ethanol. The reaction was stirred at 20°C for 18 h and concentrated, washed, then concentrated again. The crude product was purified by flash column chromatography (10% methanol / dichloromethane eluant), to yield compound **20** as a white solid (550 mg, 65%). ^{1}H NMR and LCMS confirmed purity at >98%.

^{1}H NMR (500 MHz, DMSO): δ = 5.32 (2H, s, CH_2), 6.61 (2H, s, NH_2), 6.82 (1H, dt,J=7.6, 1.0Hz, ArH), 6.93 (1H, dt J=7.6, 1.1Hz, ArH), 7.11 (2H, m, 2ArH), 7.35 (1H, dd J=7.9, 4.8Hz, ArH), 7.54 (1H, td J= J=7.8, 1.8Hz, ArH), 8.47 (1H, dd J=4.8, 1.5Hz, ArH), 8.51 (1H, d J=2.2Hz, ArH). LCMS (ES^+): m/z (%) 225 $(M+H)^+$ retention time 0.6 min. HRMS (ES^+): calcd for $C_{13}H_{13}N_4$ [M+H]+ 225.1135, found 225.1130.

1-(Naphthalen-1-ylmethyl)-1H-benzo[d]imidazol-2-amine (21)[7]

Prepared as for compound **7**, starting from 2-aminobenzimidazole (**2**) (500 mg, 3.76 mmol), potassium hydroxide flakes (317 mg, 5.64 mmol) and 1-(bromomethyl)naphthalene (873 mg, 3.95 mmol) in 10 ml ethanol. The reaction was stirred at 20°C for 18 h and concentrated, washed, then concentrated again. The crude product was purified by flash column chromatography (6% methanol / dichloromethane eluant), to yield compound **21** as a beige solid (570 mg, 55%). ^{1}H NMR and LCMS confirmed purity at >98%. ^{1}H NMR (500 MHz, DMSO): δ = 5.76 (2H, s, CH_2), 6.53 (2H, s, NH_2), 6.59 (1H, d J=7.3Hz, ArH), 6.76 (1H, m, ArH), 6.90 (1H, d J=7.8Hz, ArH), 6.94 (1H, dt J=7.8, 1.1Hz, ArH), 7.20 (1H, d J=7.7Hz, ArH), 7.36 (1H, m, ArH), 7.61 (1H, ddd J=7.9, 6.9, 1.0Hz, ArH), 7.64 (1H, ddd J=8.4, 6.9, 1.4Hz, ArH), 7.84 (1H, d J=8.2Hz, ArH), 7.99 (1H, d J=8.4Hz, ArH), 8.23 (1H, d J=8.4Hz, ArH). LCMS (ES^+): m/z (%) 274 $(M+H)^+$ retention time 0.8 min. HRMS (ES^+): calcd for $C_{18}H_{16}N_3$ [M+H]+ 274.1339, found 274.1326.

1-(Naphthalen-2-ylmethyl)-1H-benzo[d]imidazol-2-amine (22)

Prepared as for compound **7**, starting from 2-aminobenzimidazole (**2**) (500 mg, 3.76 mmol), potassium hydroxide flakes (317 mg, 5.64 mmol) and 2-(bromomethyl)naphthylene (873 mg, 3.95 mmol) in 15 ml ethanol. The reaction was stirred at 20°C for 18 h and concentrated, washed, then concentrated again. The crude product was purified by flash column chromatography (6% methanol / dichloromethane eluant), to yield compound **22** as a cream coloured solid (810 mg, 79%). ^{1}H NMR and LCMS confirmed purity at >98%.. ^{1}H NMR (500 MHz, DMSO): δ = 5.41 (2H, s, CH_2), 6.60 (2H, s, NH_2), 6.80 (1H, dt J=7.6, 1.0Hz, ArH), 6.91 (1H, dt J=7.6, 1.1Hz, ArH), 7.08 (1H, d J=7.7Hz, ArH), 7.15 (1H, d J=7.7Hz, ArH), 7.36 (1H, dd J=8.5, 1.7Hz, ArH), 7.49 (2H, m, 2ArH), 7.70 (1H, bs, ArH) , 7.83 (1H, m, ArH) , 7.89 (2H, m, 2ArH). LCMS (ES$^+$): m/z (%) 274 $(M+H)^+$ retention time 0.9 min. HRMS (ES$^+$): calcd for $C_{18}H_{16}N_3$ [M+H]+ 274.1339, found 274.1330.

***N*-(1-(3,4-Dichlorobenzyl)-1H-benzo[d]imidazol-2-yl)acetamide (23)**

1-(3,4-dichlorobenzyl)-1H-benzo(d)imidazol-2-amine (**11**) (150 mg, 0.514 mmol) was taken into ethylacetate (10 ml), and acetic anhydride (115 mg, 1.13 mmol) was added dropwise . The reaction was stirred at 20°C for 18 h and then heated to 60°C for 2 h. On cooling the reaction a white solid crystallised out, this was collected by filtration, and washed with ethylacetate (2x5 ml) to yield compound **23** as a white solid (52 mg, 30%). ^{1}H NMR and LCMS confirmed purity at >98%. ^{1}H NMR (500 MHz, DMSO): δ = 2.11 (3H, s, CH_3), 5.33 (2H, s, CH_2), 7.17 (3H, m, 3ArH), 7.38 (1H, m, ArH), 7.54 (1H, bs, ArH), 7.60 (2H, d J=8.3Hz, 2ArH), 10.63 (1H, bs, NH). LCMS (ES$^+$): m/z (%) 335 $(M+H)^+$ retention time 1.1 min. HRMS (ES$^+$): calcd for (2xCl^{35}) $C_{16}H_{13}Cl_2N_3O$ [M+H]+ 334.0508, found 334.0516.

4-Chloro-1-(3,4-dichlorobenzyl)-1H-benzo[d]imidazol-2-amine (24)

Prepared as for compound **7**, starting from 7-chloro-1H-benzo[d]imidazol-2-amine (**41**) (200 mg, 1.19 mmol), potassium hydroxide flakes (100 mg, 1.79 mmol) and 3,4-dichlorobenzyl chloride (244 mg, 1.25 mmol) in 15 ml ethanol. The reaction was stirred at 20°C for 48 h and concentrated, washed, then concentrated again. The product was purified by flash column chromatography (3% methanol/

dichloromethane eluant), to yield compound **24** as white solid (60 mg, 15%). ^{1}H NMR and LCMS confirmed purity at >98%.

The relative ^{1}H NMR signal for the CH_2-dichlorobenzyl protons at 5.28 ppm, versus 5.54 ppm for compound **29**, agrees with the expected shift for a 4-substituted *N1*-benzylated benzimidazole versus a 7-substituted benzimidazole. This observed relative shift is supported by the literature.[8]

^{1}H NMR (500 MHz, DMSO): δ = 5.28 (2H, s, CH_2), 6.82 (1H, t J=7.9Hz, ArH), 6.91 (2H, s, NH_2), 7.01 (1H, dd J=7.9, 0.8Hz, ArH), 7.10 (1H, m, ArH), 7.11 (1H, m, ArH), 7.50 (1H, d J=2.0Hz, ArH), 7.61 (1H, d J=8.3Hz, ArH). LCMS (ES^+): m/z (%) 327 $(M+H)^+$ retention time 0.8 min. HRMS (ES^+): calcd for (3xCl^{35}) $C_{14}H_{11}Cl_3N_3$ [M+H]+ 326.0013, found 325.9996.

4-(^{n} Propyloxy)-1-(3,4-dichlorobenzyl)-1H-benzo[d]imidazol-2-amine (25)

Prepared as for compound **7**, starting from 7-(^{n}propyloxy)-1H-benzo[d]imidazol-2-amine (**43**) (250 mg, 1.31 mmol), potassium hydroxide flakes (110 mg, 6.28 mmol) and 3,4-dichlorobenzyl chloride (269 mg, 1.38 mmol) in 10 ml ethanol. The reaction was stirred at 20°C for 48 h and concentrated, washed, then concentrated again. The product was purified by flash column chromatography (10% methanol / dichloromethane eluant), to yield compound **25** as white solid (65 mg, 14%). ^{1}H NMR and LCMS confirmed purity at >98%.

Absolute regiochemistry of compound **25** was confirmed by the relative ^{1}H NMR signal for the CH_2-dichlorobenzyl protons at 5.04 ppm, versus 5.48 ppm for compound **30**. This agrees with the expected shift for a 4-alkoxy substituted *N1*-benzylated benzimidazole versus a 7-substituted benzimidazole. This observed relative shift is supported by the confirmed regiochemistry of benzyloxy substituted compounds **26** and **31**, and the literature.[8] ^{1}H NMR (300 MHz, $CDCl_3$): δ = 1.02 (3H, t J=7.4Hz, CH_3), 1.88 (2H, m, CH_2) , 4.07 (2H, t J=6.8Hz, CH_2), 4.20 (2H, bs, NH_2), 5.04 (2H, bs, CH_2), 6.62 (1H, m, ArH), 6.91 (3H, m, 3ArH), 7.28 (1H, m, ArH), 7.32 (1H, m, ArH).

LCMS (ES^+): m/z (%) 351 $(M+H)^+$ retention time 0.9 min. HRMS (ES^+): calcd for (2xCl^{35}) $C_{17}H_{18}Cl_2N_3O$ [M+H]+ 350.0821, found 350.0805.

4-(Benzyloxy)-1-(3,4-dichlorobenzyl)-1H-benzo[d]imidazol-2-amine (26)

The crude filtrate from example **31** containing the 4-(benzyloxy)-1-(3,4-dichlorobenzyl)-1H-benzo[d]imidazol-2-amine was purified by flash column chromatography (6% methanol /

dichloromethane eluant), to yield compound **26** as white solid (450 mg, 27%). The weight balance was also collected (500 mg mixture of compounds **31** and **26**). ^{1}H NMR and LCMS confirmed purity at >98%.

Absolute regiochemistry of compound **26** was confirmed by NOESY NMR: no interaction between CH_2-dichlorobenzyl protons at 5.27 ppm (2H, s, CH_2) and the 7-OCH_2-benzyl signal at 5.25 ppm (2H, s, CH_2) was observed, indicating groups are not in close proximity. Also, the relative ^{1}H NMR signal for the CH_2-dichlorobenzyl protons at 5.27 ppm, versus 5.40 ppm for compound **31**, agrees with the expected shift for a 4-alkoxy substituted *N1*-benzylated benzimidazole versus a 7-substituted benzimidazole. This observed relative shift is supported by the literature..[8] ^{1}H NMR (300 MHz, DMSO): δ = 5.30 (2H, s, CH_2), 5.27 (2H, s, CH_2), 6.52 (2H, s, NH_2), 6.61 (1H, m, ArH), 6.74 (2H, m, 2ArH), 7.11 (1H, bd J=7.7Hz, ArH), 7.30 (1H, m, ArH), 7.37 (2H, m, 2ArH), 7.44 (3H, m, 3ArH), 7.58 (1H, d J=8.3Hz, ArH). LCMS (ES^+): m/z (%) 399 $(M+H)^+$ retention time 0.9 min.

4-Phenyl-1-(3,4-dichlorobenzyl)-1H-benzo[d]imidazol-2-amine (27)

Prepared as for compound **7**, starting from 7-phenyl-1H-benzo[d]imidazol-2-amine (**35**) (115 mg, 0.55 mmol), potassium hydroxide flakes (46 mg, 0.83 mmol) and 3,4-dichlorobenzyl chloride (113 mg, 0.58 mmol) in 8 ml ethanol. The reaction was stirred at 20°C for 48 h and concentrated, washed, then concentrated again. The product was purified by flash column chromatography (5% methanol / dichloromethane eluant), to yield compound **27** as a white solid (20 mg, 10%). ^{1}H NMR and LCMS confirmed purity at >98%.

Absolute regiochemistry of compound **27** was confirmed by NOESY NMR: no interaction between CH_2-dichlorobenzyl protons at 5.31 ppm (2H, s, CH_2) and the *ortho* ^{1}H signals of the pendant phenyl ring at 8.03 ppm (2H, d, CH) was observed, indicating groups are not in close proximity. As an x-ray crystal structure of the 7-phenyl isomer (compound **32**) was obtained, the absolute regiochemistry of compound **27** was confirmed as 4-phenyl-1-(3,4-dichloro)-1H-benzo(d)imidazol-2-amine.

^{1}H NMR (500 MHz, DMSO): δ = 5.31 (2H, s, CH_2), 6.77 (2H, s, NH_2), 6.94 (1H, t J=7.7Hz, ArH), 7.12 (1H, dd J=7.8, 1.0Hz, ArH), 7.16 (1H, dd J=8.4, 2.0Hz, ArH), 7.22 (1H, dd J=7.7, 1.0Hz, ArH), 7.29 (1H, m, ArH), 7.43 (2H, t J=7.7Hz, 2ArH), 7.52 (1H, d J=2.0Hz, ArH), 7.62 (1H, d J=8.3Hz, ArH), 8.03 (2H, m, ArH). LCMS (ES^+): m/z (%) 369 $(M+H)^+$ retention time 0.9 min. HRMS (ES^+): calcd for (2xCl^{35}) $C_{20}H_{16}Cl_2N_3$ $[M+H]^+$ 368.0716, found 368.0710.

4-Methoxy-1-(3,4-dichlorobenzyl)-1H-benzo[d]imidazol-2-amine (28)

Prepared as for compound **7**, starting from 7-(methoxy)-1H-benzo[d]imidazol-2-amine (**46**) (200 mg, 1.23 mmol), potassium hydroxide flakes (104 mg, 1.85 mmol) and 3,4-dichlorobenzyl chloride (252 mg, 1.29 mmol) in 10 ml ethanol. The reaction was stirred at 20°C for 48 h and concentrated, washed, then concentrated again. The reaction mixture was then purified by reverse phase prep HPLC (gradient: 20% acetonitrile/water to 80% acetonitrile/water, 9.2 mmol column packed with C18 silica). NB with normal phase silica chromatography no separation of isomers is seen. Unfortunately the compound **28** obtained still contains 25% contaminant of the 7-methoxy isomer (compound 33). ^{1}H NMR and LCMS confirmed purity at 75% (isomer ratio 3:1 (**28** : **33**)).

Absolute regiochemistry of compound **28** was confirmed by the relative ^{1}H NMR signal for the CH_2-dichlorobenzyl protons at 5.21 ppm, versus 5.38 ppm for compound **33**. This agrees with the expected shift for a 4-alkoxy substituted *N1*-benzylated benzimidazole versus a 7-substituted benzimidazole. This observed relative shift is supported by the confirmed regiochemistry of benzyloxy substituted compounds **26** and **31**, and the literature (ref: Chem. Pharm. Bull. 37(4)962-966). ^{1}H NMR for 4-methoxy isomer (300 MHz, $CDCl_3$): δ = 3.88 (3H, s, CH_3), 5.21 (2H, bs, CH_2), 6.42 (2H, bs, NH_2), 6.46 (1H, m ArH), 6.79 (1H, m, ArH), 6.86 (1H, m, ArH), 7.10 (1H, dd J=8.2, 1.9Hz), ArH), 7.43 (1H, m, ArH), 7.60 (1H, d J=8.2Hz, ArH). LCMS (ES$^+$): m/z (%) 323 $(M+H)^+$ retention time 0.8 min. HRMS (ES$^+$): calcd for (2xCl^{35}) $C_{15}H_{14}Cl_2N_3O$ $[M+H]^+$ 322.0508, found 322.0512.

7-Chloro-1-(3,4-dichlorobenzyl)-1H-benzo[d]imidazol-2-amine (29)

Compound **29** was obtained from the crude product mixture of reaction 24. Compound **29** was the more polar product obtained purified by flash column chromatography (10% methanol/ dichloromethane eluant), yielded compound **29** as a white solid (68 mg, 17%). ^{1}H NMR and LCMS confirmed purity at >98%.

The relative ^{1}H NMR signal for the CH_2-dichlorobenzyl protons at 5.54 ppm for compound **29**, versus 5.28 ppm for compound **24**, agrees with the expected shift for a 7-substituted *N1*-benzylated benzimidazole versus a 4-substituted benzimidazole. This observed relative shift is supported by the literature.[8]

^1H NMR (500 MHz, DMSO): δ = 5.54 (2H, s, CH_2), 6.78 (2H, s, NH_2), 6.84 (1H, dd J=8.0, 0.9Hz, ArH), 6.93 (1H, dd J=8.4, 2.1Hz, ArH), 6.97 (1H, t J=7.9Hz, ArH), 7.15 (1H, dd J=7.8, 0.9Hz, ArH), 7.34 (1H, d J=2.0Hz, ArH), 7.61 (1H, d J=8.3Hz, ArH). LCMS (ES^+): m/z (%) 327 $(M+H)^+$ retention time 0.8 min. HRMS (ES+): calcd for ($3xCl^{35}$) $C_{14}H_{11}Cl_3N_3$ [M+H]+ 326.0013, found 325.9998.

7-(^{n} Propyloxy)-1-(3,4-dichlorobenzyl)-1H-benzo[d]imidazol-2-amine (30)

Compound **30** was also obtained from the crude product mixture of reaction **25**. Compound **30** was the more polar product obtained purified by flash column chromatography (10% methanol/ dichloromethane eluant), yielded compound **30** as a white solid (90 mg, 20%). ^1H NMR and LCMS confirmed purity at >98%.

Absolute regiochemistry of compound **30** was confirmed by the relative ^1H NMR signal for the CH_2-dichlorobenzyl protons at 5.48 ppm for compound **30**, versus 5.08 ppm for compound **25**. This agrees with the expected shift for a 7-alkoxy substituted *N1*-benzylated benzimidazole versus a 4-substituted benzimidazole. This observed relative shift is supported by the confirmed regiochemistry of benzyloxy-substituted compounds **26** and **31**, and the literature.[8]

^1H NMR (300 MHz, $CDCl_3$): δ = 0.94 (3H, t J=7.4Hz, CH_3), 1.71 (2H, m, CH_2) , 4.03 (2H, t J=6.4Hz, CH_2), 4.30 (2H, bs, NH_2), 5.48 (2H, s, CH_2), 6.62 (1H, dd J=7.7, 1.1Hz, ArH), 7.08 (2H, m, 2ArH), 7.13 (1H, m, ArH), 7.35 (1H, s, ArH), 7.42 (1H, d J=8.3Hz, ArH). LCMS (ES^+): m/z (%) 351 $(M+H)^+$ retention time 0.9 min. HRMS (ES^+): calcd for ($2xCl^{35}$) $C_{17}H_{18}Cl_2N_3O$ $[M+H]^+$ 350.0821, found 350.0815.

7-(Benzyloxy)-1-(3,4-dichlorobenzyl)-1H-benzo[d]imidazol-2-amine (31)

Prepared as for compound **7**, starting from 7-(benzyloxy)-1H-benzo[d]imidazol-2-amine (**37**) (1g, 4.18 mmol), potassium hydroxide flakes (325 mg, 6.28 mmol) and 3,4-dichlorobenzyl chloride (858 mg, 4.39 mmol) in 15 ml ethanol. The reaction was stirred at 20°C for 48 h. The resulting precipitated product was filtered off to yield compound **31** as white solid (350 mg, 21%). ^1H NMR and LCMS confirmed purity at >98%.

Absolute regiochemistry of compound **31** was confirmed by NOESY NMR: a strong interaction between CH_2-dichlorobenzyl protons at 5.40 ppm (2H, s, CH_2) and the 7-OCH_2-benzyl signal at 5.08

ppm (2H, s, CH_2) was observed, indicating close proximity of these substituents. Also, the relative ^{1}H NMR signal for the CH_2-dichlorobenzyl protons at 5.40 ppm, versus 5.27 ppm for compound 31, agrees with the expected shift for a 7-substituted *N1*-benzylated benzimidazole versus a 4-substituted benzimidazole. This observed relative shift is supported by the literature.[8]

Compound **26** (along with a second batch of compound **31**) was obtained from the filtrate by flash column chromatography.

^{1}H NMR (500 MHz, DMSO): δ = 5.08 (2H, s, CH_2), 5.40 (2H, s, CH_2), 6.50 (2H, s, NH_2), 6.61 (1H, dd J=6.6, 2.3Hz, ArH), 6.75 (2H, m, 2ArH), 7.13 (1H, dd J=8.4, 2.0Hz, ArH), 7.32 (1H, m, ArH), 7.39 (2H, bt J=7.5Hz, 2ArH), 7.47 (3H, m, 3ArH), 7.61 (1H, d J=8.3Hz, ArH). LCMS (ES^+): m/z (%) 399 $(M+H)^+$ retention time 0.9 min. HRMS (ES^+): calcd for (2xCl35) $C_{21}H_{18}Cl_2N_3O$ [M+H]+ 398.0821, found 398.0832.

7-Phenyl-1-(3,4-dichlorobenzyl)-1H-benzo[d]imidazol-2-amine (32)

Compound **32** was obtained from the crude product mixture of reaction **27**. Compound **32** was the more polar product obtained purified by flash column chromatography (10% methanol / dichloromethane eluant), collected as a white solid (35 mg, 18%). ^{1}H NMR and LCMS confirmed purity at >98%.

Absolute regiochemistry of compound **32** was confirmed by NOESY NMR: a strong interaction between CH_2-dichlorobenzyl protons at 4.88 ppm (2H, s, CH_2) and the *ortho* proton signals of the pendant phenyl ring at 7.12 ppm (2H, d, CH) was observed, indicating close proximity of these substituents. Also an x-ray crystal structure of compound **32** in the PTR1 enzyme was obtained, confirming the absolute regiochemistry of compound **32** as 7-phenyl-1-(3,4-dichloro)-1H-benzo(d)imidazol-2-amine.

^{1}H NMR (500 MHz, DMSO): δ = 4.88 (2H, s, CH_2), 6.37 (1H, dd J=8.3, 2.1Hz, ArH), 6.50 (1H, m, ArH), 6.60 (2H, s, NH_2), 6.61 (1H, dd J=7.5, 1.1Hz, ArH), 7.00 (1H, t J=7.7Hz, ArH), 7.12 (2H, bd J=8.3Hz, ArH), 7.21 (1H, dd J=7.8, 1.1Hz, ArH), 7.31 (2H, t J=7.3Hz, 2ArH), 7.38 (2H, m, 2ArH). LCMS (ES^+): m/z (%) 369 $(M+H)^+$ retention time 0.8 min. HRMS (ES^+): calcd for $C_{20}H_{16}Cl_2N_3$ $[M+H]^+$ 368.0716, found 368.0708.

7-Methoxy-1-(3,4-dichlorobenzyl)-1H-benzo[d]imidazol-2-amine (33)

As above (example **28**), compound **33** was obtained by reverse phase prep HPLC (gradient: 20% acetonitrile/water to 80% acetonitrile/water, 9.2 mmol column packed with C18 silica). Compound **33** was the second isomer to elute off the HPLC column, and was obtained with ^{1}H NMR and LCMS purity at >99% (as 7-methoxy stereoisomer). Compound **33** was obtained as a white solid, yield 10 mg. Absolute regiochemistry of compound **33** was confirmed by the relative ^{1}H NMR signal for the CH_2-dichlorobenzyl protons at 5.38 ppm for compound **33**, versus 5.21 ppm for compound **28**. This agrees with the expected shift for a 7-alkoxy substituted *N1*-benzylated benzimidazole versus a 4-substituted benzimidazole. This observed relative shift is supported by the confirmed regiochemistry of benzyloxy-substituted compounds **26** and **31**, and the literature.[8]

^{1}H NMR (300 MHz, $CDCl_3$): δ = 3.77 (3H, s, CH_3), 4.40 (2H, bs, NH_2), 5.38 (2H, s, CH_2), 6.54 (1H, d J=7.4Hz, ArH), 7.08 (3H, m, 3ArH), 7.22 (1H, bs, ArH), 7.31 (1H, d J=8.3Hz, ArH). LCMS (ES^+): m/z (%) 323 $(M+H)^+$ retention time 0.8 min. HRMS (ES^+): calcd for (2xCl^{35}) $C_{15}H_{14}Cl_2N_3O$ $[M+H]^+$ 322.0508, found 322.0516.

1-(3,4-Dichlorobenzyl)-5,6-dimethyl-1H-benzo[d]imidazol-2-amine (34)

Prepared as for compound **7**, starting from 5,6-dimethyl-2-aminobenzimidazole (**1**) (500 mg, 3.13 mmol), potassium hydroxide flakes (263 mg, 4.69 mmol) and 3,4-dichlorobenzyl chloride (643 mg, 3.29 mmol) in 15 ml ethanol. The reaction was stirred at 20°C for 18 h and concentrated, washed, then concentrated again. The crude product was purified by flash column chromatography (6% methanol/ dichloromethane eluant), to yield compound **34** as a white solid (670 mg, 67%). ^{1}H NMR and LCMS confirmed purity at >98%.

^{1}H NMR (500 MHz, DMSO): δ = 2.18 (3H, s, CH_3), 2.19 (3H, s, CH_3), 5.20 (2H, s, CH_2), 6.38 (2H, s, NH_2), 6.86 (1H, s, ArH), 6.92 (1H, s, ArH), 7.09 (1H, d J=8.4Hz, ArH), 7.40 (1H, s, ArH), 7.60 (1H, d J=8.3Hz, ArH). LCMS (ES^+): m/z (%) 321 $(M+H)^+$ retention time 0.8 min.

7-Phenyl-1H-benzo[d]imidazol-2-amine (35)

To a solution of biphenyl-2,3-diamine, compound **36** (350 mg, 1.9 mmol), in acetonitrile (18 ml) and water (4 ml) was added cyanogenbromide 2M solution in acetonitrile (1.05 ml, 2.09 mmol). The reaction was stirred at 20°C for 18 h, and concentrated to remove the acetonitrile. Ethyl acetate (40 ml) and sodium bicarbonate solution (1x40 ml) were added, and the biphasic solution was transferred to a separating funnel. The organic layer was washed with water (1x25 ml), sodium bicarbonate solution (1x20 ml) and brine (2x30 ml), before being dried with magnesium sulfate and concentrated to remove ethylacetate. Crude compound **35** was purified by flash column chromatography (8% methanol/ dichloromethane eluant), yielded compound **35** as a white solid (250 mg, 63%). ^{1}H NMR and LCMS confirmed purity at >98%.

^{1}H NMR (500 MHz, DMSO): δ = 6.22 (2H, s, CH_2), 6.95 (1H, t J=7.7Hz, ArH), 7.08 (1H, m, ArH), 7.31 (1H, t, J=7.5Hz, ArH), 7.44 (1H, t J=7.6Hz, ArH), 7.90 (2H, bs, NH_2), 10.90 (1H, bs, NH). LCMS (ES^+): m/z (%) 210 $(M+H)^+$ retention time 0.6 min.

Biphenyl-2,3-diamine (36)

Prepared as for compound **47**, using 3-phenyl-2-nitroaniline, compound **40** (500 mg, 2.34 mmol), tin(II) chloride (2.2g, 11.7 mmol) in ethanol 25 ml). The reaction was heated in a microwave reactor at 140°C for 10 min then worked up as previously described. The solution was concentrated to remove ethyl acetate, affording compound **36** in quantitative yield, as a colourless gum. LCMS confirmed purity at >95%. Used as 'crude' in example **35**.

LCMS (ES^+): m/z (%) 184 $(M+H)^+$ retention time 0.5 min.

7-(Benzyloxy)-1H-benzo[d]imidazol-2-amine (37)

Prepared as for compound **35**, starting from 3-(benzyloxy)benzene-1,2-diamine, compound **38** (1.6g, 7.48 mmol), acetonitrile (30 ml), water (10 ml) and cyanogenbromide 2M solution in acetonitrile (4.12 ml, 8.23 mmol). The reaction was stirred at 20°C for 18 h, and concentrated to remove the acetonitrile. Crude compound **37** was purified by flash column chromatography (8% methanol / dichloromethane

eluant), yielded compound **37** as a white solid (1.1g, 61%). ^{1}H NMR and LCMS confirmed purity at >98%.

^{1}H NMR (500 MHz, $CDCl_3$): δ = 5.19 (2H, s, CH_2), 5.70 (2H, bs, NH_2), 6.67 (1H, m, ArH), 6.96 (2H, m, 2ArH), 7.30 (3H, m, 3ArH), 7.41 (2H, m, 2ArH). LCMS (ES^+): m/z (%) 240 $(M+H)^+$ retention time 0.5 min.

3-Benzyloxybenzene-1,2-diamine (38)

Prepared as for compound **47**, using 2-benzyloxy-6-nitroaniline, compound **39** (935 mg, 3.83 mmol), tin(II) chloride (3.63g, 19.13 mmol) in ethanol (30 ml). The reaction was heated in a microwave reactor at 140°C for 10 min then worked up as previously described. The solution was concentrated to remove ethyl acetate, affording compound **38** in quantitative yield, as a yellow gum. LCMS confirmed purity at >95%. Used as 'crude' in example **37**.

LCMS (ES^+): m/z (%) 215 $(M+H)^+$ retention time 0.5 min.

2-Benzyloxy-6-nitroaniline (39)

Prepared as for compound **42**, using 2-amino-3-nitrophenol (1g, 6.46 mmol), K_2CO_3 (1.34g, 9.69 mmol), DMF (35 ml) and benzyl bromide (1.21 mg, 7.1 mmol). The reaction was stirred at 20°C for 12 h. Crude compound **39** was purified by flash column chromatography (2% methanol / dichloromethane eluant), affording compound **39** as a pale orange solid (1.34g, 85%). ^{1}H NMR and LCMS confirmed purity at >98%.

^{1}H NMR (500 MHz, $CDCl_3$): δ = 5.17 (2H, s, CH_2), 6.47 (2H, bs, NH_2), 6.62 (1H, dd J=8.9, 7.8Hz, ArH), 6.99 (1H, bd J=7.1Hz, ArH), 7.44 (5H, m, 5ArH), 7.79 (1H, dd J=8.9, 1.2Hz, ArH). LCMS (ES^+): m/z (%) 245 $(M+H)^+$ retention time 0.4 min.

3-Phenyl-2-nitroaniline (40)

A solution of 2-phenylnitrobenzene (398 mg, 2 mmol) and *o*-methylhydroxylamine (118 mg, 2.5 mmol) in DMF (3 ml) was added dropwise (over 5 min) to a stirred suspension of $^{t}BuOK$ (672 mg, 6 mmol) and Cu(I)Cl (20 mg, 0.2 mmol) in DMF (7 ml). The reaction was stirred at 20°C for 60 min, and then quenched with ammonium chloride solution. The product was extracted into DCM, dried with

$MgSO_4$ and concentrated to remove the dichloromethane. The title compound **40** was separated from the side product (2-phenyl-4-nitroaniline) and remaining starting material by flash column chromatography (10% ethyl acetate / hexane eluant), affording compound **40** as a yellow solid (86 mg, 20%). ^{1}H NMR and LCMS confirmed purity at >98%.

^{1}H NMR (500 MHz, $CDCl_3$): δ = 6.5 (1H, dd J=7.7, 1.3Hz, ArH), 6.57 (2H, s, NH_2), 6.8 (1H, m, ArH), 6.89 (1H, d J=7.7Hz, ArH), 6.95 (1H, dt J=7.6, 1.2Hz, ArH), 7.2 (2H, m, 2ArH), 7.3 (1H, dt J=7.7, 1.6Hz, ArH), 7.53 (1H, dd J=7.9, 1.1Hz, ArH). LCMS (ES^+): m/z (%) 215 $(M+H)^+$ retention time 0.5 min.

7-Chloro-1H-benzo[d]imidazol-2-amine (41)

Prepared as for compound **35**, starting from 3-chlorobenzene-1,2-diamine, compound **42** (624 mg, 4.38 mmol) and cyanogen bromide 2M solution in acetonitrile (2.41 ml, 4.82 mmol), in acetonitrile (18 ml) and water (4 ml). The reaction was stirred at 20°C for 36 h. Crude compound **41** was purified by flash column chromatography (8% methanol / dichloromethane eluant), yielded compound **41** as a white solid (510 mg, 69%). ^{1}H NMR and LCMS confirmed purity at >98%.

^{1}H NMR (500 MHz, $CDCl_3$): δ = 6.40 (2H, bs, NH_2), 6.79 (1H, bm, ArH), 6.90 (1H, m, ArH), 7.0 (1H, dd J=7.8, 0.8Hz, ArH), 10.94 (1H, bs, NH). LCMS (ES^+): m/z (%) 168.5 $(M+H)^+$ retention time 0.5 min.

3-Chlorobenzene-1,2-diamine (42)

Prepared as for compound **47**, using 2-chloro-6-nitroaniline (1.0 g, 5.8 mmol), tin(II) chloride (5.5g, 29 mmol) in ethanol 30 ml). The reaction was heated in a microwave reactor at 140°C for 10 min then worked up as previously described. The solution was concentrated to remove ethyl acetate, affording compound **42** in quantitative yield, as a colourless gum. LCMS confirmed purity at >95%. Used as 'crude' in example **41**.

LCMS (ES^+): m/z (%) 143 $(M+H)^+$ retention time 0.5 min.

7-(^{n}Propyloxy)-1H-benzo[d]imidazol-2-amine (43)

Prepared as for compound **35**, starting from 3-^{n}propyloxybenzene-1,2-diamine, compound **44** (410 mg, 2.47 mmol) and cyanogen bromide 2M solution in acetonitrile (1.36 ml, 2.72 mmol), in acetonitrile (12 ml) and water (3 ml). The reaction was stirred at 20°C for 36 h. Crude compound **43** was purified by flash column chromatography (8% methanol / dichloromethane eluant), yielded compound **43** as a white solid (300 mg, 64%). ^{1}H NMR and LCMS confirmed purity at >98%.

^{1}H NMR (500 MHz, $CDCl_3$): δ = 1.01 (3H, t J=7.4Hz, CH_3), 1.72 (2H, m, CH_2), 4.03 (2H, m, CH_2), 5.83 (2H, bs, NH_2), 6.46 (1H, m, ArH), 6.86 (2H, m, ArCH), 10.70 (1H, bs, NH). LCMS (ES^+): m/z (%) 192 $(M+H)^+$ retention time 0.6 min.

3-Propoxybenzene-1,2-diamine (44)

Prepared as for compound **47**, using 2-nitro-6-propoxyaniline, compound **45** (300 mg, 1.53 mmol), tin(II) chloride (1.45 g, 7.65 mmol) in ethanol (15 ml). The reaction was heated in a microwave reactor at 140°C for 10 min then worked up as previously described. The solution was concentrated to remove ethyl acetate, affording compound **44** as a pale yellow gum in quantitative yield. ^{1}H NMR and LCMS confirmed purity at >95%.

^{1}H NMR (500 MHz, $CDCl_3$): δ = 1.07 (3H, t J=7.4Hz, CH_3), 1.82 (2H, m, CH_2), 3.44 (2H, m, NH_2), 3.98 (2H, t J=6.5Hz, CH_2), 6.40 (2H, m, ArH), 6.68 (1H, t J=8.0Hz, ArH). LCMS (ES^+): m/z (%) 167 $(M+H)^+$ retention time 0.4 min.

2-Nitro-6-propoxyaniline (45)

Prepared as for compound **48**, using 2-amino-3-nitrophenol (500 mg, 3.23 mmol), K_2CO_3 (669 mg, 4.84 mmol), DMF (35 ml) and 3-bromopropane (438 mg, 3.35 mmol). The reaction was stirred at 20°C for 12 h. Crude compound **45** was purified by flash column chromatography (3% methanol/ dichloromethane eluant), affording compound **45** as a pale yellow solid (460 mg, 73%). ^{1}H NMR and LCMS confirmed purity at >98%.

^{1}H NMR (500 MHz, $CDCl_3$): δ = 1.09 (3H, s, CH_3), 1.91 (2H, s, CH_2), 4.02 (2H, m, CH_2), 6.47 (2H, m, NH_2), 6.64 (1H, dd J=8.9, 7.8Hz, ArH), 6.91 (1H, d J=7.6Hz, ArH), 7.75 (1H, dd J=8.9, 1.1Hz, ArH). LCMS (ES^+): m/z (%) 197 $(M+H)^+$ retention time 0.4 min.

7-Methoxy-1H-benzo[d]imidazol-2-amine (46)

Prepared as for compound **35**, starting from 3-methoxybenzene-1,2-diamine, compound **47** (341 mg, 2.47 mmol) and cyanogen bromide 2M solution in acetonitrile (1.36 ml, 2.72 mmol), in acetonitrile (12 ml) and water (3 ml). The reaction was stirred at 20°C for 36 h. Crude compound **46** was purified by flash column chromatography (8% methanol/ dichloromethane eluant), yielded compound **46** as a white solid (258 mg, 64%). ^{1}H NMR and LCMS confirmed purity at >98%.
^{1}H NMR (300 MHz, DMSO): δ = 3.89 (3H, s, CH_3), 5.9 (2H, s, NH_2), 6.49 (1H, m, ArH), 6.76 (2H, m, 2ArH), 10.80 (1H, bs, NH). LCMS (ES^+): m/z (%) 164 $(M+H)^+$ retention time 0.5 min.

3-Methoxybenzene-1,2-diamine (47)

To a solution of 2-methoxy-6-nitroaniline, compound **48** (500 mg, 2.98 mmol) in ethanol (15 ml) was added tin(II) chloride (2.83g, 14.9 mmol). The reaction was heated in a microwave reactor at 140°C for 10 min. The reaction mixture was poured into $NaHCO_3$ solution (30 ml) and the product was extracted into ethyl acetate (2x25 ml). The organic layers were combined and washed with sodium bicarbonate solution (1x30 ml), water (1x30 ml) and brine (2x40 ml), before being dried with MgSO4 and concentrated to remove ethyl acetate. Afforded compound **47** as a pale yellow oil in quantitative yield. ^{1}H NMR and LCMS confirmed purity at >95%.
^{1}H NMR (500 MHz, $CDCl_3$): δ = 3.60 (4H, bs, $2NH_2$), 4.03 (3H, s, CH_3), 6.58 (2H, m, 2ArH), 6.84 (1H, m, ArH). LCMS (ES^+): m/z (%) 139 $(M+H)^+$ retention time 0.4 min.

2-Methoxy-6-nitroaniline (48)

To a stirred solution of 2-amino-3-nitrophenol (500 mg, 3.23 mmol) in DMF (15 ml) was added K_2CO_3 (669 mg, 4.84 mmol) and methyl iodide (504 mg, 3.55 mmol). The reaction was stirred at 20°C for 12 h, then poured into $NaHCO_3$ solution (20 ml), and the product was extracted into ethyl acetate (2x30 ml). The organic layers were combined then washed with water (1x30 ml), sodium bicarbonate solution

(1x30 ml) and brine (2x40 ml), before being dried with $MgSO_4$ and concentrated to remove ethyl acetate. Crude compound **48** was purified by flash column chromatography (3% methanol/ dichloromethane eluant), affording compound **48** as a yellow solid (520 mg, 96%). ^1H NMR and LCMS confirmed purity at >98%.

^1H NMR (500 MHz, $CDCl_3$): δ = 3.90 (3H, s, CH_3), 6.40 (2H, bs, NH_2), 6.64 (1H, dd J=8.9, 7.8Hz, ArH), 6.91 (1H, d J=7.6Hz, ArH), 7.70 (1H, dd J=8.9, 1.1Hz), ArH). LCMS (ES^+): m/z (%) 169 $(M+H)^+$ retention time 0.4 min.

Enzyme Assays

Inhibitor sensitivity against PTR1 and DHFR were determined using the cytochrome-*c* coupled assay method.[9]. An HPLC method was used to determine the sensitivity of PTR1 to compound **32** in clarified cell lysates. Assays were carried out in 20 mM sodium citrate (pH 6.0) containing 1 mM EDTA, 1% (v/v) DMSO, 0.1% (v/v) Triton X-100 and 100 μM NADPH cofactor. *Tb*PTR1 (1.1 nM) was preincubated with varying concentrations of compound **32** (0-1 μM) for 5 min, before reactions were initiated using 25 nM dihydrobiopterin. Aliquots (100 μl) of enzymatic reactions were removed after 2 min and oxidized with iodine under alkaline conditions and analysed by HPLC as previously described.[9] The IC_{50} of **32** against native PTR1 in *T. brucei* clarified lysates (20,000 g, 30 min, 4 °C; final assay concentration 100 μg ml^{-1}) was determined in a similar fashion. Lysates were prepared[10] using *T. brucei* harvested from rats,[11] as previously described. Trypanothione reductase activity was measured in clarified lysates as previously described[12] to ensure adequate extraction of parasites.

Trypanosome Growth Assays

These were performed as previously described.[13]

References

[1] C. P. Mpamhanga, D. Spinks, L. B. Tulloch, E. J. Shanks, D. A. Robinson, I. T. Collie, A. H. Fairlamb, P. G. Wyatt, J. A. Frearson, W. N. Hunter, I. H. Gilbert, R. Brenk, *J. Med. Chem.*, **2009**, *52*, 4454-4465.

[2] D. Vlaovic, J. Canadanovicbrunet, J. Balaz, I. Juranic, D. Djokovic, K. Mackenzie, **1992**, *56*, 199-206.

[3] S. Podunavac-Kuzmanovic, S. Markov, D. Barna, **2007**, *6*, 687-698.

[4] P. Moldt, O. Axelsson, Vol. EP 0545845 (A1) **1993**.

[5] O. Axelsson, M. Thaning, P. Moldt, Benzimidazole compounds useful as calcium channel blockers., Vol. US 5314903 (A) **1994**.

[6] K. Sterz, L. Mollmann, A. Jacobs, D. Baumert, M. Wiese, **2009**, *4*, 1897-1911.

[7] A. M. Simonov, A. F. Pozharskii, **1964**, *34*, 1572.

[8] R. Iemura, M. Hori, H. Ohtaka, *Chem. Pharm. Bull.*, **1989**, *37*, 962-966.

[9] E. J. Shanks, H. B. Ong, D. A. Robinson, S. Thompson, N. Sienkiewicz, A. H. Fairlamb, J. A. Frearson, *Anal. Biochem.*, **2010**, *396*, 194-203.

[10] T. J. Vickers, A. H. Fairlamb, *J. Biol. Chem.*, **2004**, *279*, 27246-27256.

[11] S. M. Lanham, *Nature*, **1968**, *218*, 1273-1274.

[12] M. C. Jockers-Scherubl, R. H. Schirmer, R. L. Krauth-Siegel, *Eur. J. Biochem.*, **1989**, *180*, 267-272.

[13] D. Spinks, E. J. Shanks, L. A. T. Cleghorn, S. McElroy, D. Jones, D. James, A. H. Fairlamb, J. A. Frearson, P. G. Wyatt, I. H. Gilbert, *ChemMedChem*, **2009**, *4*, 2060-2069.
